# Analyzing Chronological Change in Postoperative Magnetic Resonance Imaging Results in Patients With Kienböck’s Disease by Using an Original Grading System

**DOI:** 10.7759/cureus.24178

**Published:** 2022-04-16

**Authors:** Takeshi Ogawa, Akira Ikumi, Sho Kohyama, Yuki Hara, Yuichi Yoshii, Naoyuki Ochiai, Masashi Yamazaki

**Affiliations:** 1 Department of Orthopedic Surgery, National Hospital Organization, Mito Medical Center, Ibaraki, JPN; 2 Department of Orthopedic Surgery and Sports Medicine, Tsukuba University Hospital Mito Clinical Education and Training Center, Mito, JPN; 3 Department of Orthopedic Surgery, Kikkoman General Hospital, Noda, JPN; 4 Department of Orthopedic Surgery, Faculty of Medicine, University of Tsukuba, Tsukuba, JPN; 5 Department of Orthopedic Surgery, Tokyo Medical University Ibaraki Medical Center, Ibaraki, JPN

**Keywords:** ordinal scale, quantitative analysis, proton density weighted image, magnetic resonance imaging, necrotic lunate, kienböck’s disease

## Abstract

Background and objective

Signal changes in MRI for Kienböck’s disease have only been qualitatively assessed so far. In light of this, we proposed a new grading system for quantitative analysis with an ordinal scale.

Methods

The study included 31 patients (17 men, 14 women) with Kienböck's disease. By referring to Nakamura’s MRI grading system, we devised a grading system with five grades (Grades 1-5) using proton density-weighted (PDW) coronal images with respect to the signal intensity of the lunate. All cases were examined by using the MRI grading system by three hand surgeons, both preoperatively and postoperatively. We evaluated the inter-rater reliability of our grading system by using the interclass correlation coefficient. After surgery, we implemented annual MRI evaluation for as long as possible and quantitatively assessed changes in MRI grades. We also investigated the correlation between postoperative MRI grades, Mayo Wrist Scores (MWS), and age at the surgery by using Pearson’s coefficient.

Results

The MRI evaluation was performed 2-15 years after surgery. The reliability of our grading system was high; inter-rater interclass correlation coefficients were 0.783 (examiners 1-2), 0.780 (examiners 1-3), and 0.825 (examiners 2-3), representing a substantial agreement. The correlation coefficient between the MRI grade and MWS was -0.31, suggesting a mild negative correlation; postoperative MRI grade also correlated with age at surgery (Pearson’s coefficient: 0.447).

Conclusions

Our proposed MRI grading system has high reliability and could be used to assess the regeneration of a necrotic lunate for quantitative analysis on an ordinal scale. Improvements were observed one to four years postoperatively, demonstrating a mild correlation with the clinical results.

## Introduction

Kienböck’s disease was first described in 1910 by radiologist Robert Kienböck, who described radiographic changes associated with lunate malacia [1]. The exact mechanism of Kienböck’s disease is not yet fully understood. It is believed to be an interaction between altered vascular perfusion, repeated microtrauma, changes in lunate bone structure, changes in loading and movement, and potential systemic disease [2]. Signal changes during MRI evaluation for Kienböck’s disease have been qualitatively assessed [3]. In order to properly diagnose Kienböck’s disease and its progression, it is necessary to establish a standardized test based on MRI analysis. In this study, we developed a new evaluation method by using an ordinal scale that can be classified into five levels according to the degree of disease progression based on MRI signal intensity. MRI analysis by using this ordinal scale is expected to assess the effectiveness of the treatment and progress of necrosis. In patients with Kienböck’s disease, we performed a combined therapy that included non-concentrated bone marrow transfusion, low-intensity pulsed ultrasound, and external fixation [3,4]; however, this treatment did not result in better outcomes than other treatments [5-9]. There are some questions that remain unanswered regarding this method, such as when is the improvement of MRI signal after surgery observed, whether the improvement is continuous, and whether MRI signal improvement correlates with clinical results or patients’ age. In this study, we, therefore, assessed the validity of the grading system, quantitatively evaluated postoperative MRI changes over time, and evaluated the correlation between MRI signal improvement and clinical results following our procedure for Kienböck’s disease.

## Materials and methods

**Figure 1:**
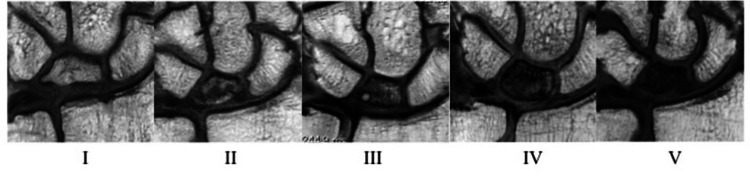
MRI grading system using the T1 or PDW coronal image On this scale, with respect to the signal intensity of the lunate, Grade I was considered almost normal; Grade II indicated localized regions of slightly decreased signal intensity; Grade III indicated a slight, generalized decrease in signal intensity; Grade IV represented low signal intensity with regions of high or isointense signals, and Grade V indicated generalized low signal intensity MRI: magnetic resonance imaging; PDW: proton density-weighted

Our study involved 31 patients (17 men, 14 women) with Kienböck’s disease by using Ogawa’s procedure [3,4]. This retrospective study was approved by our hospital’s ethical committee (R02-106), and written informed consent was obtained from all participants. Our indications for surgical intervention were diagnosis of Kienböck’s disease at stages II, IIIa, and IIIb. Preoperative Lichtman stages observed were as follows: stage II in nine patients, stage IIIa in 16 patients, and stage IIIb in six patients; the mean age at the time of surgery was 43.6 (16-78) years. A 1.5- or 3-T magnetic resonance apparatus (Gyroscan NT Intera, Philips Medical Systems, Amsterdam, Netherlands), with a small-diameter surface coil (Philips Medical Systems), was used for MRI evaluation [10]. We referred to Nakamura’s MRI grading system [11] to devise an individual grading system using proton density-weighted (PDW) coronal images. On this scale, with respect to the signal intensity of the lunate, Grade I was considered almost normal; Grade II indicated localized regions of slightly decreased signal intensity; Grade III indicated a slight, generalized decrease in signal intensity; Grade IV represented a low signal intensity with regions of high or isointense signals; and Grade V indicated a low, generalized signal intensity (Figure 1).

Grades were determined by using the most severe coronal slice on MRI. Using the MRI grading system, all cases were both preoperatively and postoperatively examined by three hand surgeons. We then evaluated the inter-rater reliability of the grading system by using the interclass correlation coefficient. After surgery, we implemented an annual MRI evaluation and assessed the changes in MRI grade on an ordinal scale. We also investigated the correlation between MRI grade at the final follow-up, Mayo Wrist Score (MWS) [12], and age at the time of surgery, by using Pearson’s coefficient.

Statistical analysis

We evaluated the inter-rater reliability of our MRI grading system by using the interclass correlation coefficient. We evaluated the correlation between the clinical results, MWS, and age using Pearson’s correlation coefficient; the significance level was set at p=0.05. All statistical analyses were performed with Bellcurve for Excel version 3.20 (SSRI Co., Tokyo, Japan).

## Results

**Figure 2:**
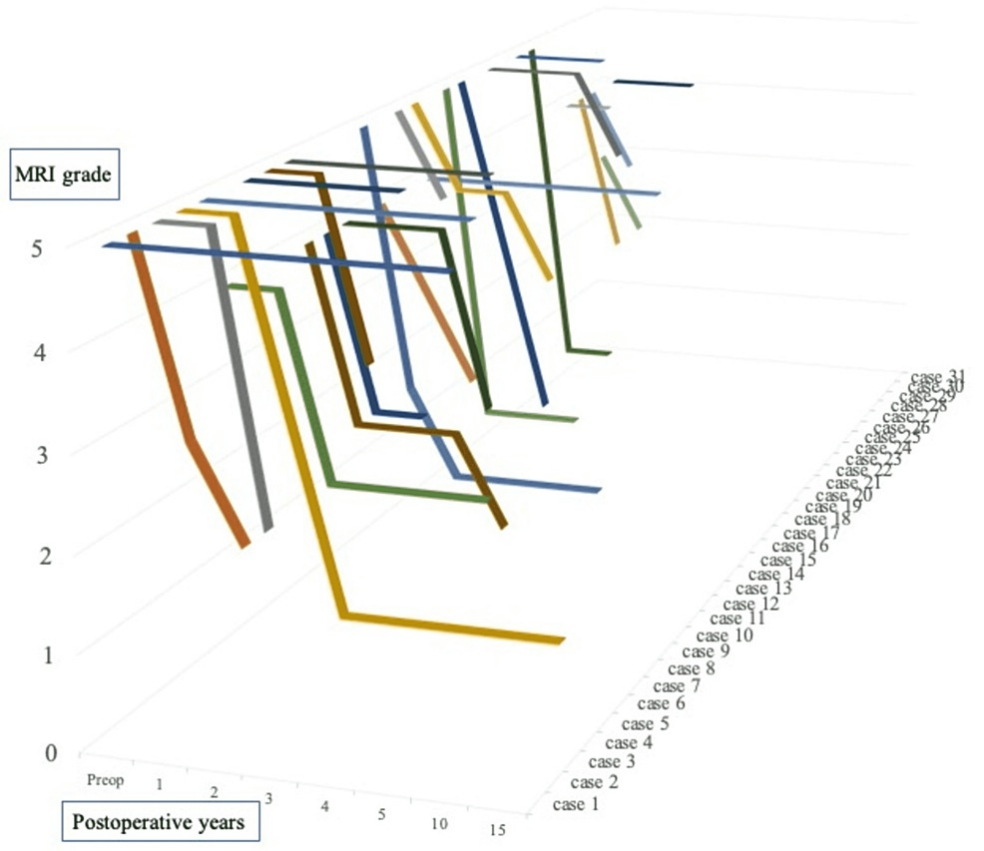
Chronological change in postoperative MRI grade for all patients MRI: magnetic resonance imaging

MRI evaluation was performed for 2-15 years after surgery. The reliability of our MRI grading system was high, with inter-rater interclass correlation coefficients of 0.783 (examiners 1-2), 0.780 (examiners 1-3), and 0.825 (examiners 2-3), representing a substantial or near-perfect agreement. Improvements were observed one to four years postoperatively (Figure 2), from an average grade of 4.5 (range: 3-5) preoperatively to 2.75 (range: 1-5) at the final follow-up.

**Figure 3:**
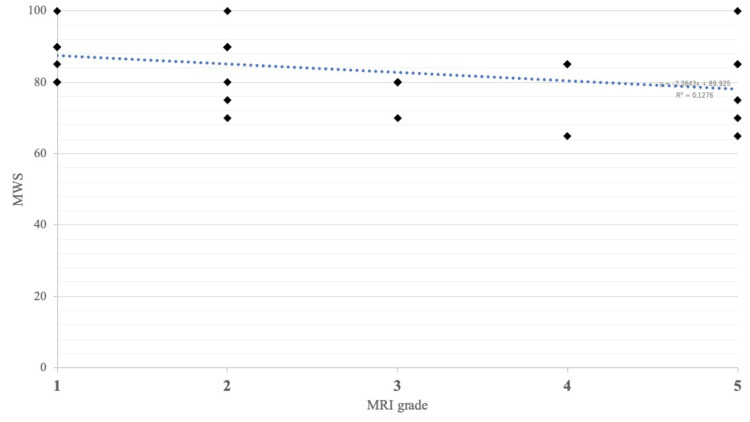
Pearson’s coefficient between MWS and MRI grades Pearson’s coefficient was -0.315 MRI: magnetic resonance imaging; MWS: Mayo Wrist Score

Improved MRI signal intensity was observed in 23 patients (74%): 13 cases within one year, six patients after one to two years, and four patients after more than two years. To date, none of the improved patients have deteriorated since experiencing improvement. A satisfactory improvement in MRI Grade I or II was observed in 17 patients (74%); additionally, there were no cases in which the recovered intensity of the lunate worsened. The average MWS at the last follow-up was 83.4 (65-100), and the Pearson’s coefficient was -0.315, which slightly correlated with the MRI grade at the last follow-up (Figure 3).

**Figure 4:**
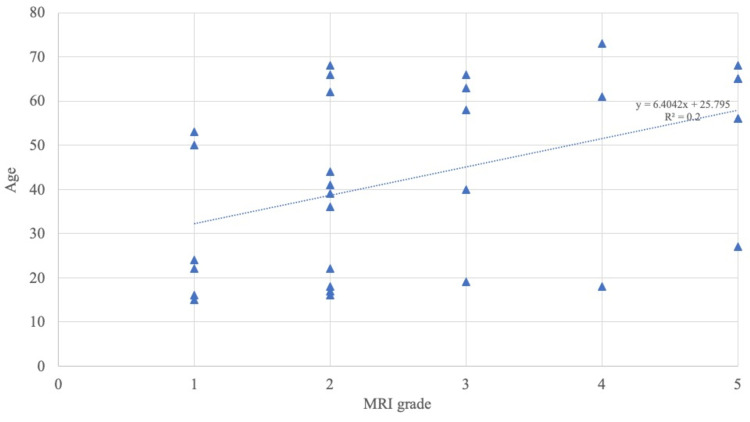
Pearson’s coefficient between age at the surgery and MRI grade at the final follow-up Pearson’s coefficient was 0.447 MRI: magnetic resonance imaging

The postoperative MRI grade also correlated with age at the time of surgery (Pearson’s coefficient: 0.447) (Figure 4).

By contrast, there was no correlation between MWS and age (Pearson’s coefficient: 0.068).

**Figure 5:**
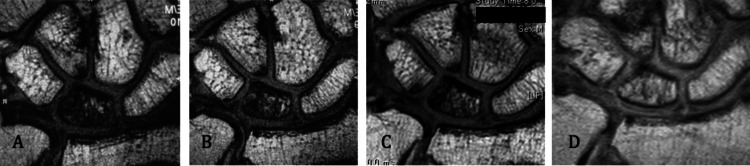
Chronological change in the postoperative MRI results of a 41-year-old man before and after surgery for Kienböck’s disease A. Preoperative magnetic resonance image (Grade IV). B. One year postoperatively (Grade IV). C. Two years postoperatively (Grade IV). D. Three years postoperatively (Grade II) MRI: magnetic resonance imaging

The representative case involved a 41-year-old man who underwent surgery. His preoperative MRI Grade was IV, which improved to Grade IV at one year, Grade IV at two years, and Grade II at five years postoperatively (Figures 5A-5D). He reported no pain at three months postoperatively, and his MWS was 90 points at one year postoperatively; therefore, MRI was not performed at three and four years postoperatively.

## Discussion

We investigated the chronological change in postoperative MRI results in 31 patients with Kienböck’s disease by using a novel grading system. The validity of the grading system was assessed and changes in postoperative MRI over time were evaluated on an ordinal scale. Improved MRI signal intensity was observed in 23 patients, with changes appearing within one year in 13 patients. Furthermore, the MRI grade exhibited a slight correlation with the clinical results.

Very few reports have quantitatively evaluated the MRI findings in Kienböck’s disease. Nakamura et al. [11] classified the appearance of the lunate; its signal intensity was graded on a scale of I to V, and they evaluated T1- and T2-weighted images both preoperatively and postoperatively. Imaeda et al. [13] also concluded that the intensity of the signal of the lunate on T2-weighted images indicates the severity of the disease, with a decreased signal containing a high spot or an increased signal suggesting revascularization. Conversely, Ogawa et al. [10] described that PDW (T1-weighted) MRI accurately reflects the vascular status of the lunate, as evidenced by comparison with histological analyses of lunate specimens. They also reported that in the fast-field echo (T2-weighted) images, there were no correlations with histopathological observations.

T2-weighted images reflect various changes, including bone marrow edema, blood vessels, and bleeding. We, therefore, attempted to quantitatively evaluate lunate regeneration using the PDW images. By contrast, Schmitt et al. [14] described gadolinium-enhanced MRI-based classification to delineate the pattern of osteonecrosis in different parts of the lunate bone; middle reparative zone; and distal, normal, viable lunate bone. Based on the signal intensity in different zones of the lunate on MRI, Schmitt et al. classified three stages of Kienböck’s disease: stage A was "Marrow edema with viable and intact bony trabeculae", stage B was "Early marrow necrosis with fibrovascular reparative tissue", and stage C was ‘Necrotic bone marrow with collapse [14]". We also considered the best way to investigate the recovery from bone avascular necrosis using both gadolinium-enhanced MRI and PDW imaging. Gadolinium-enhanced MRI may reflect blood flow and may be useful in combination with PDW, which reflects bone regeneration, to assess treatment efficacy and stage. This is one of the limitations of the present study. Consequently, the reliability of our classification was high, demonstrating a slight correlation with the clinical results. Nakamura et al. [11] reported that there was no significant correlation between lunate signal intensity and clinical outcomes. Although there were good clinical outcomes in our case, some patients exhibited poor MRI recovery, which is another limitation of this study. Additionally, we believe that considering the correlation between MRI and clinical outcomes, it may be useful to employ gadolinium-enhanced MRI together with our MRI grading system.

Regarding the age of patients with Kienböck’s disease, van Leeuwen et al. [15] described age as a risk factor for lunate collapse, while Koh et al. [16] reported that the younger generation had better radiological improvement, implying more revascularization of the lunate. The correlation between age and MRI improvement remains unclear, and there are no reports pertaining to age and MRI improvement in the literature. Matsui et al. [17] reported that younger patients had a tendency toward improvement in MRI findings; however, no significant differences in the clinical outcomes, regardless of whether MRI improvement was achieved, were observed. In our study, there was a significant difference between older and younger patients in terms of MRI improvement (Figure 4); however, our study included nine women in menopause. The conditions of Kienböck’s disease may differ between younger and older individuals; hence, our data remain theoretical.

## Conclusions

Our proposed MRI grading system demonstrated high reliability and could be used to assess the regeneration of the necrotic lunate on an ordinal scale. Using this MRI ordinal scale, the correlation between the clinical outcomes after treatment for Kienböck’s disease was recognized, and improvements in MRI grade were observed one to four years postoperatively. Furthermore, the recovered intensity of the lunate remained at the same level as the intensity.
